# Mr. Carmichael, on Carbonate of Iron in Cancer

**Published:** 1807-03

**Authors:** Richard Carmichael

**Affiliations:** North Cumberland Street, Dublin


### Medical and Phyſical Journal.

VOL. XVII.] MARCH, 1807. [NO. 97.

*Printed for R. PHILLIPS, by W. Thorne, Red Lion Court, Fleet Street, London.*

### *To the Editors of the Medical and Physical Journal.*

GENTLEMEN,

SINCE the publication of my Essay on the Effects of Carbonate of Iron upon Cancer, I have tried other preparations of that metal; and desirous of extending, as soon as possible, any advantage that may be derived from my additional experience of its efficacy, I take advantage of your widely circulated Journal to publish a few observations, that I am presuming enough to think may be of some little service; and, happily, I am enabled to detail some cases, in addition to those already related, in which there could not be a shadow of doubt with respect to the nature of the disease, or good effects of the remedy.

It is chiefly in ulcerated cancers that the preparations of iron are found serviceable, as the remedy can in them be brought into immediate contact with the diseased part; but in occult cancers, the impossibility of applying it in the same manner, renders it of course less efficacious. Yet in one lady, Miss G. who consulted me on account of a hard tumour in her right breast, accompanied with lancinating pains, the most decided benefit was derived from the external as well as internal use of this remedy. It was applied externally by means of cloths moistened in a mixture of equal parts of tincture of muriated iron and water; and internally, the precipitated carbonate of iron, or tartarized iron, was exhibited in such doses as the stomach could bear without uneasiness. The pains under this treatment entirely subsided, and the hardness in the breast became far less obvious.

In every case of this disease, wherever situated, the cancer was chiefly composed of a substance neither jelly nor cartilage, but in a certain degree approaching the consistence of both; in some cases more closely resembling the former, in others the latter.

With respect to this substance, which is truly the Cancer, I shall mention a circumstance perhaps not hitherto noticed, viz. that it may be cut, compressed, or punctured, without producing any sensation in the patient. This fact seems to favour the opinions of those who would support the independent life of cancer; but I do not introduce it with that view, for with readiness I shall resign an hypothesis, which at present appears to me to explain the phenomena of this disease with more consistency than any hitherto offered, whenever stronger arguments or closer analogies shall more truly explain its nature and cause. I may say with truth, I entertain no prejudice in its favour, if I except an excusable partiality for the guide that led me to the trial of a remedy which will be of extensive advantage to mankind, at a period when the theory which induced its discovery may be forgotten.

I was lately led, from a consideration that the iron in the blood is united with the phosphoric acid, to try the effects of the phosphate and oxy-phosphate of iron; but at present, I am determined not to theorize, and will therefore reserve every thing but facts till another opportunity, although I confess the restraint I impose upon myself, by withholding some curious observations connected with this part of my subject.

I have found those preparations, but particularly the oxy-phosphate, infinitely more efficacious than the other salts of iron upon cancerous ulcers.

The phosphate of iron is readily prepared by mixing a solution of phosphate of soda with a solution of sulphate of iron; the phosphate of iron, of a dark blue colour, falls to the bottom, and is afterwards to be separated by filtering. But the oxy-phosphate cannot be obtained without much greater difficulty; and I have particularly to regret the want of a trading chemist in this city, as I have neither time nor opportunity to furnish myself with the quantity I would constantly require. But as I have already sufficient testimony of the efficacy of this preparation, in order to make it known to the public, I think I should be culpable in delaying any longer to give that information which may induce others to prosecute the interesting inquiry.

Of the immediate effects of the phosphate of iron in alleviating or removing altogether the characteristic lancinating pains of cancer, I shall briefly mention two cases of that disease, in its most advanced stage. In August last I was desired to see Miss R——, a lady between forty and fifty fifty years of age, possessing that peculiar paleness of countenance so characteristic of cancer. The disease was at the time in its most advanced stage, a deep and frightful ulcer, with elevated and everted edges, extending from the axilla to the sternum, and discharging in great abundance a thin fœtid matter; the induration extended as far as the clavicle, and downwards upon the false ribs, it also filled the entire of the axilla. Her strength was so reduced, that she could not rise from her chair without assistance, and she particularly complained of the severity of the lancinating pains, which however she was accustomed to bear in silence, but their attack was always known to her friends by the sudden start or shuddering their violence excited.

Mr. M'Mullen, apothecary, in Capel-street, occasionally visited her, and prescribed anodyne medicines in such doses as might afford her some relief, for several surgeons of eminence, whom she had consulted long before I saw her, informed her friends of the hopeless nature of her disorder. I could not do otherwise than concur in their opinion; but although I had not the slightest hopes of her recovery, I had little doubt in affirming that the pains would be much alleviated by sprinkling the ulcer daily with a chalybeate preparation.

On the 7th of August, 1806, the cavity of the ulcer was nearly filled with phosphate of iron; over this was placed lint, and above all a pledget spread with a mild ointment. Two days afterwards, she declared that the "blue powder" had so much relieved her, that she scarcely felt a pain, and distinguished her present sensation as a slight stinging.

She was dressed in the same manner daily till the 1st of September, without any return of the pains, and with an evident amendment in the appearance of the ulcer, so that her friends began to entertain sanguine hopes of her recovery; but her strength was evidently declining, on account of the profuseness of the discharge from the ulcer, the entire surface of which betrayed no kind of feeling on being cut or pricked with a sharp instrument.

At this period I fancied that the discharge might have been increased by the application of the powder, it was therefore laid aside for four or five days, but she herself begged that it might be resumed, for the pains were beginning to return with their former violence, and the ulcer now put on a more pale and ashy colour.

The powder was again resorted to, as I did not find that

 the the discharge was diminished during its intermission, and on its resumption the pains again vanished. But her powers of life gradually declined, the arm of the affected side became œdematous, and swelled to her fingers' ends; a symptom which I witnessed, sooner or later, in every cancer of the breast which terminated fatally; and on the 12th of September, without a return of the lancinating pains, she died.

I shall not extend this paper, by relating the history of the other case; it is sufficient to observe, that it was an ulcerated cancer of the breast of a woman about 40, and that the induration extended into the axilla, the discharge being profuse, with frequent hæmorrhage, and the lancinating pains so violent that she could not suppress her cries whenever they attacked her.

It being Dr. Geoghegan's month of attendance at the Dispensary, I requested him to observe with me the effects of the phosphate of iron, which was applied on the 1st of September, in such a manner as to fill the entire cavity of the ulcer. The next day she declared that the pains were so much alleviated since the application, that she passed an easier night than she had done for many months before, and that she slept soundly; a comfort which she had not latterly enjoyed.

She was this day dressed with lint, as Dr. Geoghegan wished to ascertain beyond a doubt, whether the alleviation of the pain was owing to chance, or the agency of the chalybeate; but the next day she complained that the shooting pains had returned with their accustomed violence. It would extend this paper beyond its necessary limits, were I to copy the notes I took each day of her case; suffice it to say, that to determine the powers of the medicine, she was dressed three days successively with the phosphate of iron, and the next three with lint; that, on such days as the powder was applied, the pains disappeared, and on the succeeding days they returned, beginning with a stinging sensation, and gradually increasing to their former severity.

This experiment was frequently made, and always with the same result. During this treatment the discharge continued very profuse, the appearance of the ulcer varied considerably, for the pale ashy colour of the fungus which arose from the surface of the ulcer, became at first of a bright red, and from time to time sloughed away, having first changed to a dark liver colour. When I perceived it approaching this state, I removed with the knife as much as as would not bleed upon cutting; and on each section, branches of the white cancerous substance were apparent, ramifying through the liver-coloured fungus, now dead, and beginning to separate. By these means, in about six weeks, the scirrhous substance was diminished more than one half; but the patient's strength began to decline, perspirations at night and a profuse discharge from the ulcer combining to reduce her; yet her appetite (a circumstance not a little extraordinary) was voracious, and she had to the last hour a continual craving for food; but at length, worn out by the profuseness of the discharge, she died on the 6th of November.

Though these two cases terminated fatally, yet no man of candour will think the medicine disparaged for not being an elixir vitæ. If it were only capable of alleviating the severe pains of this disorder, it would still be an invaluable remedy; but, happily, its effects are not confined to such cases; and I have had a more pleasing proof in the complete recovery of Mrs. R. This lady came from the county of Westmeath, to place herself under my care, in May last, by the advice of her physician, Dr Barlow, a gentleman with whom I have not the honour of being personally acquainted, but who, by publishing the case of this lady while under his care, and her present state of health since her return to his neighbourhood, would very much contribute to remove that scepticism which inevitably attends the promulgation of such uncommon facts.

On the 7th of May, 1806, I first saw my patient; she was considerably advanced in years, and of a delicate constitution. The disease, whose first appearance she observed twelve months before, was situated in her right breast; her attention being excited to it by pains shooting from a small lump about the size of a hazel nut. The tumour gradually increased in size, and the pains in frequency and severity; at length her breast ulcerated, and a large fungus protruded considerably beyond the integuments. She had been using, by the advice of Dr. Barlow, for some time before she came to Dublin, the carbonate of iron, both internally and externally, which, she said, had alleviated the pains, and at the same time improved her general health, which had considerably suffered. This lady remained three months under my care, at the end of which time she returned to the country with every appearance of restored health; the diseased parts having separated, the ulcer healed, the pains vanished, and no remaining hardness to be discovered.

 I took

I took accurate Notes of the progress of her recovery which at another time shall be given to the public, as their relation would swell this paper beyond the limits adapted for your Journal; I shall therefore now only mention, that for the first six or seven weeks the ulcer was dressed with precipitated carbonate of iron, and at the same time large doses of that medicine were taken internally; that during the remainder of the time Mrs. R. continued under my care, the ulcer was in general dressed with oxy-phosphate of iron, of a white colour in general. I mention this, because I had not always that preparation in readiness; *and in Dublin there was no person from whom I could obtain it.* During this treatment I did not hesitate to remove with the knife or scissars, such part of the diseased mass as did not discharge any blood upon being divided, or shew any sign of sensation under the knife. Thence the remedy was always brought into immediate contact with the part upon which it was to act, and the cure was the more quickly advanced, by not waiting for the more tedious separation of such parts as were deadened by the action of the remedy.

I have repeatedly since heard from this lady, and have the satisfaction to find that she continues in perfect health, without any symptom whatever of a return of her former complaints.

I might relate several other cases of cancerous ulcers of the face, lip, nose, and other parts of the body, which became completely well under the use of the above remedy; but as there is justly so much scepticism with regard to matters so novel, I have only selected those prominent cases, which admit neither of doubt nor cavil; and will also serve the purpose of demonstrating, that I have no desire to insinuate that the remedy recommended will, in every instance, effect a cure. This would be a species of folly, which could only bring discredit on that which, I trust, will be found a most useful medicine. It can only be expected to cure, where the disease has not too far extended its ravages. Mercury can do no more in syphilis, nor any medicine in any case.

In my Essay I have stated facts, inferences from which must favour, in a very strong degree, the idea that carcinoma is *a true animal fungus* or *parasitical production,* affording, in its origin and growth, a strong analogy to the vegetable fungus, which springs from the bark and leaves of plants, and being, in both kingdoms, no farther connected with the living body in which it is placed, than as it it derives from thence its proper nidus and nourishment, which last it assimilates by its own innate powers, and not by any continuation of vessels from the one to the other. I have also ventured to conjecture, that the iron which modern chemistry has discovered to be present in the bodies of animals possessing red blood, has not been provided merely to impart the red colour to that fluid, but to answer more important purposes in the animal œconomy, one of which most probably is, to prevent the formation of those animal fungi or parasitic productions in the superior classes of animals, to the generation of which their complex structure would probably render them more liable, were not this antidote provided.

These opinions are strongly supported by some experiments, in which I found that all the oxides of iron are destructive to the lives of white-blooded animals, but that they possess this power in different degrees, the oxy-phosphate greatly exceeding every other. If three common earth worms, as nearly as possible of equal size and vivacity, were thrown into three different portions of carbonate, phosphate, and oxy-phosphate of iron, either in the state of powder or suspended in water, I always found the last ten times more powerful than the phosphate, and 30 times more powerful than the carbonate ; the worm acted on by this preparation being in general killed in less than six minutes, while that in the phosphate lived for an hour, and the one in the carbonate three hours.

This property of the oxides of iron, by which they are destructive to the lives of white-blooded animals, may render it of the greatest utility in a variety of ways, but particularly as a remedy for intestinal worms, of which the tænia still continues difficult of cure, but will scarcely baffle the effects of the oxy-phosphate of iron.

I cannot conclude without adverting to a striking coincidence between this oxy-phosphate and the secret remedy mentioned in the Dissertation of Triller, as well with respect to its appearance, as the excitement it occasioned, and the general progress of the cure under its use. In this Dissertation * we are informed, that " the celebrated Petrus

* Tale tamen remedium a Boerhaavio jure desideratum, nulloære mercabile, nullo inquam, auro estimabile, habuisse unice rarissima quidem felicitate, videtur celeberrimus iste sævi cancri domitor, uti vocabatur, Petrus Alliot, Barroducæus, *rerum medicarum et chemicarum scientissimus,* qui generosioris cujusdam feminæ mamillæ sinistra crudellissima cancri teterrimi trus Alliot, a man of great medical and chemical knowledge, recovered a woman of rank from a cancer, which had eaten away, in a dreadful manner, her left breast. He daily sprinkled the ulcer with a white powder, whose component parts he never disclosed. In less than an hour from the application, a degree of excitement ensued, (levis coorta febricula) which however soon ceased, and left the patient at ease. By a perseverance in the use of this powder for six weeks, the lips of the wound changed from a livid to a red colour, and the watery ichor into healthy concocted matter, after which the wound quickly healed."

"Such (the author continues) are the words of a person who relates the transaction, not from hearsay, but the evidence of his own eyes; and it deserves the more credit as he was deeply skilled in medicine and chemistry, and was no less than the celebrated Olaus Borrichius. He published them in Actis Med. Hafniens. Vol. i. Obs. 72, p. 160, edited by Thom. Bartholine. The cure made much noise at Paris, and was considered rather as a miracle than a natural occurrence. Theophilus Bonetus afterwards transferred the account to his System of Medicine; but he regrets with Borrichius, that the Inventor of a powder so capable of being of advantage to mankind, should be so much more covetous of perishable profit than of immortal fame, that he suffered the secret to die and be buried with him."

The terrimi pertinacissimque tabe per integrum jamjam quadriennium, immane quantum peresæ inspersit quotidie *albicantem quemdam pulvisculum* sibi soli notum, unde post horulæ spatium, *levis coorta febricula*, quæ tamen mox cessavit, et quietem ægræ indulsit. Ita continua pulveris hujus inspersione, sesquemense, effecit ut *vulneris labra ex livido*, in *rubicundulum* terminarentur colorem, et *ichor serosus paulatim* in *coctum laudabiliter pus verteretur:* quo facto, vulgaribus sarcoticis, ministrante chyrurgo, plagam consolidavit.—Quæ sunt ipsa verba testis non auriti, aut rerum harum imperiti, sed oculate potius præsentis, et qui visa sibi loquitur, ut ipse scribit, simulque rerum medicarum as chemicarum juxta peritissimi, hincque tanto magis fidem plenam merituri, nimirum doctissimi illius et celeberrimi per orbem viri, Olai Borrichii, qui hæc ipsa suo tempore, Parisiis, publice gesta, miraculo tamen propiora, quam rei naturali, aut ordinariæ, candide, docte, copiose, atque simul ornate, memoriæ prodidit, in Actis Med. Hafniens. per Thom. Bartholinum, virum summum, publicatis, Vol. I. Obs. 72, p. 160. Ex quibus eamdem mirabilem Historiam in Medicinam suam septentrionalem transtulit Theophil. Bonetus. Tom. II. Lib. iv. Sect. 11. Obs. 6. p. 158. Vehementer autem cum eodem laudato Borrichio, dolendum, illum ipsum, admirandæ virtutis pulverem, seculo tantopere pro futurum, cum suo auctore, lucri perituri cupidione, quam famæ æternum mansuræ, jamdudum in pulverem ciniremque abiisse.

*Halleri Disput. Chirurg. Tom. II. p.* 492.

The identity of these remedies is a conjecture, the similarity of their beneficial effects is none: one has been lost to the world by the selfishness of its discoverer; but in spite of the selfishness of the world, by which is always understood a very narrow portion of it, the other never shall be lost. Silent and tedious may be its progress to established reputation; yet the credit it has already obtained, inspires me with a perseverance and pride, which no other cause could have awakened. From experience convinced of its value, and impressed with the strongest confidence that the benefits of my discovery will one day or other be extensively felt and acknowledged, I shall patiently bear the strictures and opposition which my opinions may meet with, satisfied with the conviction, that no neglect or opposition of a beneficial disclosure can subvert its utility, or obstruct its extension.

I am, &c.

RICHARD CARMICHAEL.

*North Cumberland Street, Dublin,*
*Jan.* 12, 1807.

